# Interleukin-17 and inflammatory bowel disease: a 2-sample Mendelian randomization study

**DOI:** 10.3389/fimmu.2023.1238457

**Published:** 2023-11-17

**Authors:** Yangke Cai, Xuan Jia, Liyi Xu, Hanwen Chen, Siyuan Xie, Jianting Cai

**Affiliations:** ^1^ Department of Gastroenterology, The Second Affiliated Hospital, Zhejiang University School of Medicine, Hangzhou, Zhejiang, China; ^2^ Zhejiang University Cancer Institute, Key Laboratory of Cancer Prevention and Intervention, China National Ministry of Education, The Second Affiliated Hospital, Zhejiang University School of Medicine, Hangzhou, China

**Keywords:** inflammatory bowel disease, interleukin-17, Mendelian randomization, multivariable Mendelian randomization, genetic epidemiology

## Abstract

**Introduction:**

Observational studies have discovered a contradictory phenomenon between interleukin-17 (IL-17) and inflammatory bowel disease (IBD). The study aimed to confirm the causal association between each subtype of IL-17 and IBD.

**Methods:**

We performed a 2-sample univariable and multivariable mendelian randomization (MR) to determine which subtype of IL-17 is causally related to IBD and its subtypes, and used a series of sensitivity analysis to examine the reliability of the main MR assumptions.

**Results:**

We found that IL-17B, IL-17E and IL-17RB were significantly associated with an increased risk of UC (IL-17B: OR: 1.26, 95% CI, 1.09-1.46, P < 0.01; IL-17E: OR: 1.17, 95% CI, 1.05-1.30, P < 0.01; IL-17RB: OR: 1.30, 95% CI, 1.20-1.40, P < 0.0001) while IL-17C and IL-17RC showed causal effects on the increased risk of CD (IL-17C: OR: 1.23, 95% CI, 1.21-1.26, P < 0.0001; IL-17RC: OR: 2.01, 95% CI, 1.07-3.75, P=0.03). The results of multivariable MR (MVMR) showed that the causal effects of IL-17B and IL-17E on UC were unilaterally dependent on IL-17RB, while the effects of IL-17C and IL-17RC on CD were interdependent.

**Discussion:**

Our study provided new genetic evidence for the causal relationships between each subtype of IL-17 and IBD, promoting future mechanistic research in IBD.

## 1 Introduction

Inflammatory bowel disease (IBD), consisting of ulcerative colitis (UC) and Crohn’s disease (CD), is an immune-mediated disease characterized by chronic intestinal inflammation. The pathogenesis that triggers the onset of IBD mainly depends on the interaction among genetic, environmental and microbial factors (1, 2). Currently, IBD is a global disease. Its evolution consists of four stages: Emergence, Acceleration in Incidence, Compounding Prevalence and Prevalence Equilibrium. The Western world is now in the third epidemiological stage, which causes a very large economic burden (3). Concerning this health problem, there has been an expansion in IBD therapeutic options. Conventional treatment for IBD includes aminosalicylates (5-ASA), corticosteroids (CSs), and immunomodulators (4). Given this phenomenon, there is an urgent need to discover novel drug targets for the treatment of IBD. New treatments include biologics, such as anti-TNFα agents (including infliximab, adalimumab, certolizumab pegol and golimumab), anti-integrin agents (vedolizumab), anti-IL-12/23 p40 subunit agents (including ustekinumab, risankizumab, mirikizumab) and new targeted small molecules (including upadacitinib, ozanimod, filgotinib), which have revolutionized the treatment of IBD (5–7). However, take the anti-TNF agents for example, approximately one-third of patients with IBD fail to react to anti-TNF therapy. Even 20–40% of initial responders lose their response over time, suggesting the need for new drugs (8). Given this phenomenon, there is an urgent need to discover novel drug targets for the treatment of IBD.

Cytokines, such as interleukins 1, 6, 17, 22, 23, TNF, and IFN, have been reported to be directly engaged in the pathogenesis of IBD (9). Among these cytokines, IL-17 is a controversial cytokine involved in chronic inflammation and is worthy of investigation. It consists of 6 ligands (IL-17A to IL17F) and 5 receptors (IL-17RA to IL-17RE). To date, IL-17A inhibitors have emerged over the past decades as effective treatments for ankylosing spondylitis (AS) and psoriatic arthritis (PsA) (10, 11). Although IBD, AS and PsA are all immune-mediated diseases, some studies have reported that patients with IBD treated with IL-17A inhibitors experience a considerable number of exacerbations (12). A clinical trial revealed that secukinumab, an IL-17A antagonist, has been related to worsened symptoms compared to placebo in CD patients (13). There are no definitive studies showing a clear association between IBD and any IL-17 subtype. Thus, our study attempts to determine the role of other subsets of IL-17 in IBD.

Mendelian randomization (MR) is a powerful tool that examines a causal association between an exposure and an outcome by using genetic variants (14). To examine the role of IL-17 and its subtypes in IBD, we systematically searched protein quantitative trait loci (pQTL) for MR analysis. The pQTL can reflect the protein levels in the patient population, and they can be used as instruments for MR analysis. Multivariable MR (MVMR) is an extension of MR that is used to assess whether the associations exist after controlling for potential confounders. Compared to observational studies, MVMR enables us to account for potential confounding factors and enhance the robustness of our findings (15). In the present study, we used two-sample univariable and multivariable MR analyses to investigate the causal effect of IL-17 (including IL17A-F and IL-17RA-RD) on IBD (including CD and UC).

## 2 Methods

### 2.1 Study design

**Figure 1:**
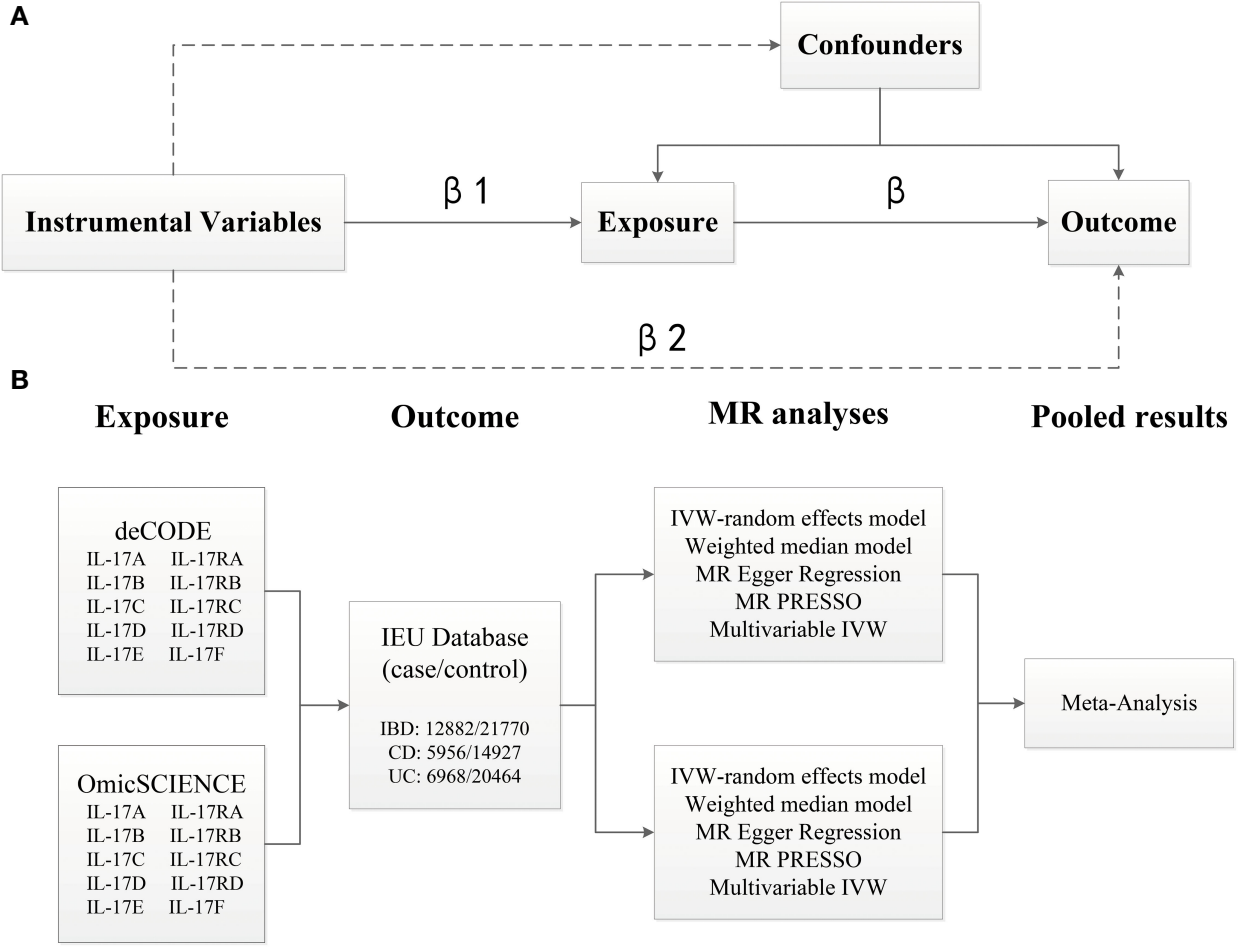
Overview of the analytical plan. **(A)** Three principal assumptions of Mendelian Randomization (MR). Specifically, the genetic instrumental variables should be associated with exposure, and be associated with outcome only via exposure, and not be associated with any measured or unmeasured confounding factors. **(B)** Univariable and multivariable MR to estimate the causal relationships of IL-17 (OmicSCIENCE and deCODE databases) on IBD (EBI database). MR analyses were conducted per database and were subsequently meta-analyzed to generate pooled estimates.

We performed two‐sample univariable MR to estimate the causal relationships of IL-17 (including IL-17A, IL-17B, IL-17C, IL-17D, IL-17E, IL-17F, IL-17RA, IL-17RB, IL-17RC and IL-17RD) on IBD (including CD and UC) based on genome-proteome–wide association studies for IL-17 from the OmicSCIENCE and deCODE databases and GWAS summary statistics for IBD from the European Bioinformatics Institute [EBI] databases. Then, we performed a meta-analysis by pooling the calculated effect for each outcome together to improve the power statistics. Finally, we performed multivariable MR on IL-17 subtypes that exhibited significant effects on the same outcome to determine their direct effects on IBD (**Figure 1**). The study was designed and reported in compliance with the STROBE-MR statement (**Supplementary Material**).

### 2.2 Data sources

The summary statistics of pQTLs for IL-17 were obtained from the OmicSCIENCE and deCODE databases. The OmicSCIENCE study comprised 4,775 human plasma proteins assayed by the SomaScan v4 platform among 10,708 individuals (16), whereas the deCODE study linked association of 27.2 million sequence variants and 373 diseases and other traits to the levels of 4,907 plasma proteins measured in 35,559 Icelanders (17). Detailed information on the participants, gene expression measurements and genotyping has been described on the website. We extracted the summary-level statistics for IBD from the IEU database (18) [cases/controls for IBD: 25,042/34,915; CD: 12,194/28,072; UC: 12,366/33,609]. Based on radiological, endoscopic, and histopathological evaluations, all included cases met the clinical diagnostic criteria for IBD.

### 2.3 Selection of genetic instrumental variants

The genome-wide significance level of p < 5×10^-8^, a minor allele frequency <0.01 and a clumping algorithm with a cutoff of r2 = 0.001 and kb = 10000 were used to avoid linkage disequilibrium (LD). The strength of the genetic variants of each exposure was estimated by R2 and F statistics. The IVs with F-statistics over 10 were used for subsequent analyses to avoid weak instrument bias (19). To prevent potential pleiotropy, the IVs were further searched using PhenoScanner V2 (http://www.phenoscanner.medschl.cam.ac.uk/) to evaluate whether the instrumental variables were directly associated with IBD or known confounding factors (i.e., smoking).

### 2.4 Statistical analyses

The inverse-variance weighted (IVW) meta-analysis with multiplicative random model was used as the major analysis for causal estimation. Cochran’s Q test was performed to detect heterogeneity among the genetic variants. The weighted median model, MR−Egger regression model and Mendelian randomization pleiotropy residual sum and outlier [MR-PRESSO] were performed as sensitivity analyses and to examine the existence of horizontal pleiotropy that violated the main MR assumptions. Among these, the weighted median method is utilized to strengthen causal estimates when up to half of the genetic instruments included are invalid (20). MR−Egger regression and MR-PRESSO were applied to detect and correct for pleiotropic effects (21). To maximum the power of our analyses, we performed a meta-analysis to combine the IVW causal estimates with fixed-effect mode from the OmicSCIENCE dataset and deCODE dataset. The results are presented as odds ratios (ORs) with 95% confidence intervals (CIs) and can be interpreted as the average change in the outcome for each unit increase in the level of the respective exposure. The power statistics were calculated using mRnd (22).

Analyses were performed in R [v. 4.2.1] statistical software. The two-sample univariable and multivariable MR analyses were performed using the package ‘TwoSampleMR’ [0.5.6] and ‘MRPRESSO’ [1.0]. The meta-analyses were conducted with the ‘meta’ package [5.5.0] (23). The P values in this study were 2-sided, and values < 0.05 were deemed significant. For MVMR analysis, a conservative Bonferroni-correction was performed to account for multiple test.

## 3 Results

### 3.1 Genetic instruments for IL-17

The summary statistics of genetic instrumental variables for subtypes of IL-17 are presented in **Supplementary Tables 1**, **2**. The F statistics of the selected variables were all above 20, suggesting that there was no strong evidence for weak instrument bias (**Supplementary Tables 1**, **2**). After searching the PhenoScanner database, SNPs were removed for being associated with IBD and its known confounders (**Supplementary Table 3**).

### 3.2 Causal effects of IL-17 on IBD

**Figure 2:**
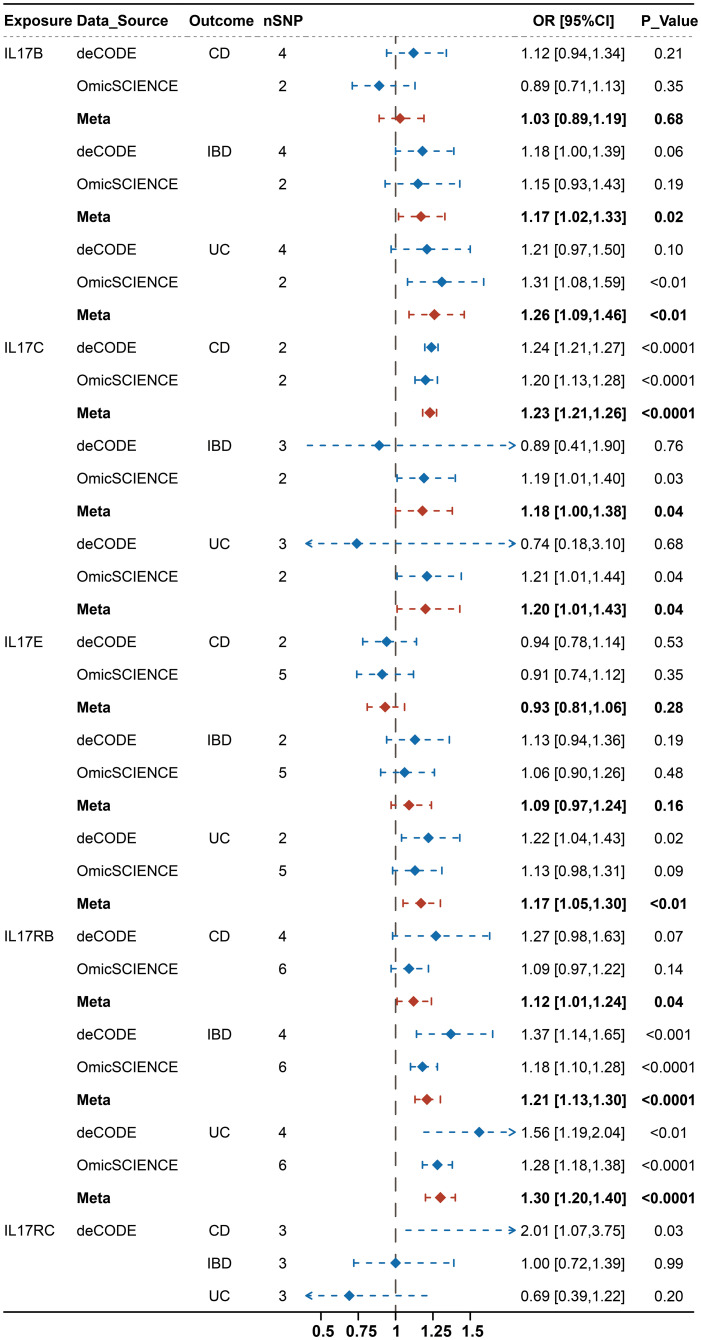
Integrated causal IVW estimator from a fixed effect meta-analysis for the causal effect of genetically predicted IL-17(B, C, E, RB, RC) on IBD (IBD, CD, UC) before MR-PRESSO.

The results of the causal association between IL-17 and IBD are presented in **Figure 2**. The meta-analyses of estimates from IVW suggested that IL17B, IL17C, IL17E, IL17RB and IL17RC were all causally associated with one subtype of IBD, but their results were inconsistent (**Supplementary Table 4**). For example, IL-17B, IL-17E and IL-17RB were significantly associated with an increased risk of UC (IL-17B: OR: 1.26, 95% CI, 1.09-1.46, P < 0.01; IL-17E: OR: 1.17, 95% CI, 1.05-1.30, P < 0.01; IL-17RB: OR: 1.30, 95% CI, 1.20-1.40, P < 0.0001) rather than CD. On contrast, IL-17C and IL-17RC showed causal effects on the increased risk of CD (IL-17C: OR: 1.23, 95% CI, 1.21-1.26, P < 0.0001; IL-17RC: OR: 2.01, 95% CI, 1.07-3.75, P = 0.03). However, IL-17C also showed weak effects on UC (OR: 1.20, 95% CI, 1.01-1.43, P = 0.04). To further adjust for potential and interaction confounders, we reassessed the causal associations between IL-17 and IBD after correcting the outliers by MR-PRESSO. Interestingly, the most results remained consistent results in terms of direction of magnitude while the causal effect of IL-17C on UC turned into insignificant (**Supplementary Figure 1**).

**Figure 3:**
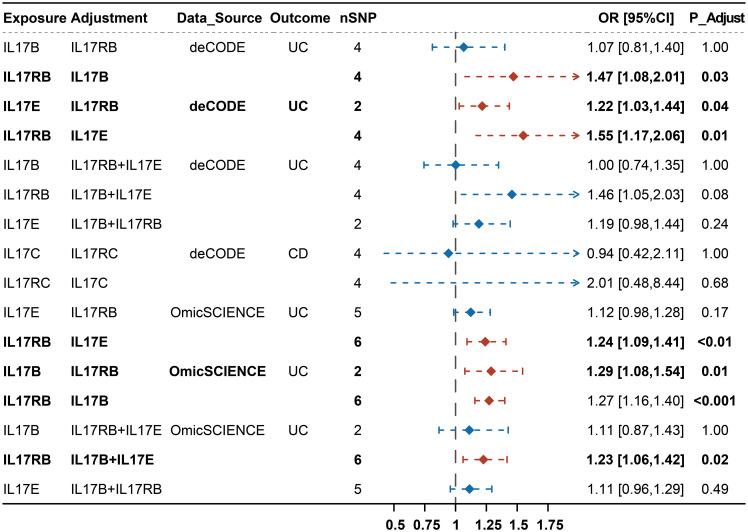
Causal effect of genetically predicted IL-17 (B, C, E, RB, RC) on CD and UC using MVMR.

We then estimated mutually the effects of IL-17B, IL-17E and IL-17RB on UC, and IL-17C and IL-17RC on CD using MVMR and performing Bonferroni correction for multiple test. We observed that the effects of IL-17B, IL-17E and IL-17RB on UC were not exactly consistent and IL-17B and IL-17E tended to be non-significant after correcting for the effect of IL-17RB, whereas IL-17RB tended to show significant effect on UC after correcting for IL-17B and IL-17E separately or simultaneously. We also observed that the effects of IL-17C and IL-17RC on CD turned into insignificant after adjusting for the effect of each other (**Figure 3**; **Supplementary Table 5**). The above results indicated that the effects of IL-17B and IL-17E on UC may be mainly through IL-17RB while the causal effect of IL-17RB on UC may not be completely dependent on IL-17B and IL-17E. Moreover, the effects of IL-17C and IL-17RC on CD were interdependent.

### 3.3 Sensitivity analyses

Cochran’s Q test, MR Egger regression model and MR-PRESSO analyses all showed that the main estimates of IL-17 on IBD were hardly affected by any heterogeneity and pleiotropy. After correcting for the limited outliers detected by MR PRESSO, the main results were consistently with previous results in terms of direction and magnitude. Moreover, the weighted median and MR-Egger analyses were consistent with the IVW results, indicating the robustness of the results (**Supplementary Table 3**). The power statistics of the significant results were provided in **Supplementary Table 6**. For the significant results, the power statistics of the effect of IL-17E and IL-17RB on CD and IL-17RC on CD was all over 0.85 while IL-17B on UC was 0.67 and IL-17C on CD was 0.57, which was relatively lower than we expected. However, to test the reliability of the results, we re-estimate the above associations using more relaxing criteria of selecting IVs (P=5×10^-6^, R2 = 0.001, KB=10000) and found that the results were consistent and the power statistics of the effect of IL-17B on UC was 0.93 and IL-17C on CD was 0.79, demonstrating the reliability of the results. In addition, the leave-one-out sensitivity analysis confirmed that there was almost no significant change in the risk estimations for genetically predicted IL-17 levels and IBD risk after removing 1 SNP at a time, except for the estimations of IL-17RB on UC and IL-17RC on CD from deCODE database (**Supplementary Figures 2**-**17**). However, these SNPs were not detected by MR-PRESSO as outliers and there were no heterogeneity and pleiotropy as mentioned above, we believe the results were reliable. A scatter plot was used to visualize the effect size of each MR method (**Supplementary Figures 2**-**17**).

## 4 Discussion

In the present study, we identified that higher levels of IL-17B, IL-17E and IL-17RB were possibly associated with a higher risk of UC while higher levels of IL-17C and IL-17RC were possibly associated with a higher risk of CD. MVMR analysis results showed that the causal effects of IL-17B and IL-17E on UC were unilaterally depended on IL-17RB, while the effects of IL-17C and IL-17RC on CD were interdependent.

The association between IL-17 and IBD has been extensively investigated in recent studies. Numerous studies have demonstrated a contradictory relationship between IL-17 and IBD. Knowledge about the contribution of IL-17 cytokines to the pathogenesis of IBD is limited and mostly focuses on IL-17A. For example, a systematic review including 3 human studies and 6 animal studies found that IL-17A could be a protective factor for IBD (12, 24). Recently, Lee et al. constructed acute model of gut injury and found IL-17A provided the protective effects to intestinal mucosa via Act-1 (25). Moreover, Bruno Emond et al. reported that the risk of new-onset IBD among patients with chronic inflammatory diseases exposed to IL-17A blockers increased based on the MarketScan Research databases (26). However, Yin et al. constructed a DSS-induced colitis model in mice and reported that blocking IL-17A can inhibit DSS-induced UC (27). Besides, a nationwide cohort study including 16,793 patients reported that patients with PsO and PsA/AS who were treated with an IL-17A blocker did not have a higher risk of IBD (28). In view of the contradictory phenomenon, our study aims to elucidate the possible causal effects of the other subtypes of IL-17 on IBD and provide new insights for future basic and clinical studies.

Among the subtypes of IL-17, except IL-17A, the function of other subtypes including IL-17B, IL-17C, IL-17E et al. in IBD remain largely elusive. Although these factors have not been studied fully in IBD, their role in other autoimmune diseases or inflammatory diseases may also have reference value for our results. Many studies have reported that IL-17B is pathogenic in inflammatory arthritis and gastroenterology cancers (29, 30), and the expression levels of IL-17B are significantly higher in patients of systemic sclerosis than in controls (31). IL-17E, also named IL-25, is a key element of type 2 immune responses, a driver of inflammatory diseases, and interacts with IL-17RB (32). In 2021, XKH001, the first IL-17E inhibitor developed by Dong Chen et al, was approved for use in patients with refractory or drug-resistant inflammatory diseases worldwide and is currently in phase I clinical study (33). The expression of IL-17C was found to be increased in psoriatic skin and atopic eczema, representing a promising target for the treatment of psoriasis and atopic eczema (34, 35). IL-17RC has also been found to enhance proinflammatory function in kidney disease by regulating CD4+ T cells. Combined with our present results, among all subtypes of IL-17, we speculate that IL-17RB blocker (including IL-17B and IL-17E inhibitors) and IL-17C inhibitor may have the potential therapeutic value in treating UC and CD, respectively.

For the purpose of assessing our findings’ reliability, we conducted a series of sensitivity analyses. MR-PRESSO, MR Egger and leave-one-out analyses were used, and the results were robust. These results indicated that treatments targeting IL-17B, IL-17C, IL-17E, IL-17RB and IL-17RC could be candidates for IBD therapy. According to PhenoScanner and the GWAS catalog, there was no evidence of pleiotropy, indicating that our results were plausible. Among the strengths of our analysis are the meta-analysis, which we performed to enhance the reliability of the results, and the large sample size that was used. Another strength is the approach that we assessed the causal effects of IL-17 and its subtypes on the development of IBD using a 2-sample MR approach and MVMR analysis. The approach is less susceptible to confounding and reverse causation than observational studies. It is worth mentioning that we also used the GWAS of deLange et al. as an outcome for validation and found that the causal effect of IL-17E on UC is still consistent with the current results (**Supplementary Tables 7**, **8**), suggesting the causal effect of IL-17E on IBD is more reliable than others, and further enhance the potential therapeutic value of XH001 on UC. Several limitations should also be acknowledged. Firstly, the GWAS datasets used in this study were based on the European population, which prevents the results from being applied to all races. Secondly, we investigated the causal relationship between IL-17 and IBD only at the protein level. The source of IL-17 we obtained was serum rather than intestinal tissue. Thirdly, the leave-one-out analysis showed that there are SNPs that influence the final result when estimating the causal effect of IL-17RB on UC and IL-17RC on CD from deCODE database, which means that part of the final results was not robust enough. However, we have to emphasize that this study is a comprehensive study integrating deCODE and OmicSCIENCE databases to investigate the causal effects on IBD using two-sample MR and MVMR. We have performed multiple sensitivity analyses and didn’t detect significant heterogeneity and pleiotropy. Overall, despite a slight flaw, we believe that our results are still reliable. Fourthly, because of the unavailability of individual data, our analysis may have potential bias, such as lifestyle factors and drug interference. Fifthly, based on current knowledge, there may be unknown confounding factors associated with SNPs. Finally, to have a point identification (one effect estimates) for this local average treatment effect, one must also assume that every individual would have the same potential response to that change in genomic variant (monotonicity assumption).

## 5 Conclusion

In conclusion, our study suggests that IL-17B, IL-17E and IL-17RB are causal risk factors for UC, and IL-17C and IL-17RC are causal risk factors for CD. These findings promote the future mechanistic study in IBD. However, researches on these cytokines in IBD are still immature and more studies are needed.

## Data availability statement

The original contributions presented in the study are included in the article/**Supplementary Material**. Further inquiries can be directed to the corresponding authors.

## Author contributions

JC and SX designed the study and reviewed the literature. YC and XJ performed the data analysis and wrote the manuscript for the study. LX and HC contributed to the reviewing of the literature. All authors contributed to the article and approved the submitted version.
