# Supplementary material for: Interleukin-17 and inflammatory bowel disease: a 2-sample Mendelian randomization study
Source: Front Immunol. 2023 Nov 17;14:1238457. doi: 10.3389/fimmu.2023.1238457 (PMC10690942; doi:10.3389/fimmu.2023.1238457)

Figure S1 Integrated causal IVW estimator from a fixed effect meta-analysis for the causal effect of genetically predicted IL-17(B, C, E, RB, RC) on IBD (IBD, CD, UC) after MR-PRESSO.

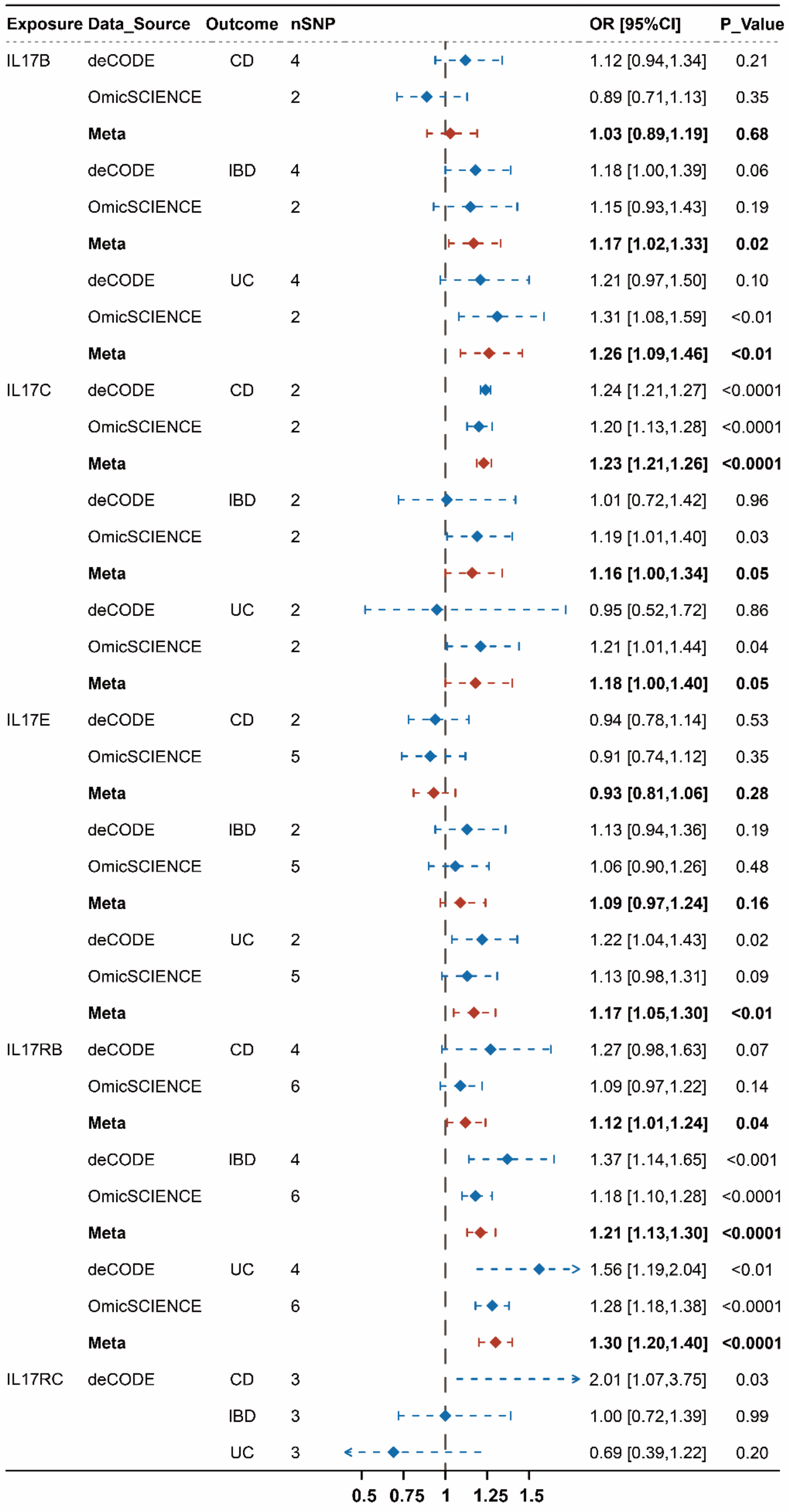

FigureS2. Scatter plot (A-C) of the causal effect of IL-17A on IBD (UC, CD) in OmicSCIENCE dataset.

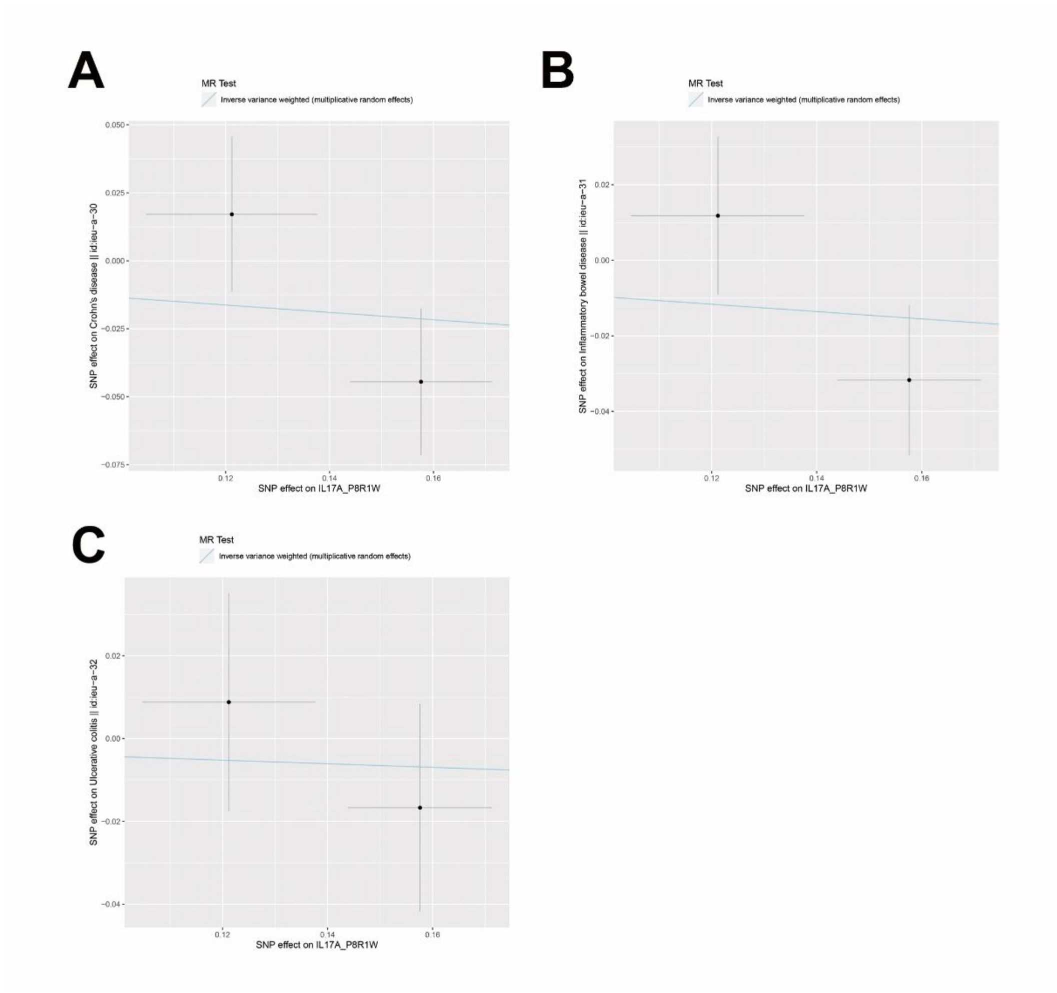

FigureS3. Leave-one-out analysis (A, C, E), and scatter plot (B, D, F) of the causal effect of IL-17B on IBD (UC, CD) in deCODE dataset.

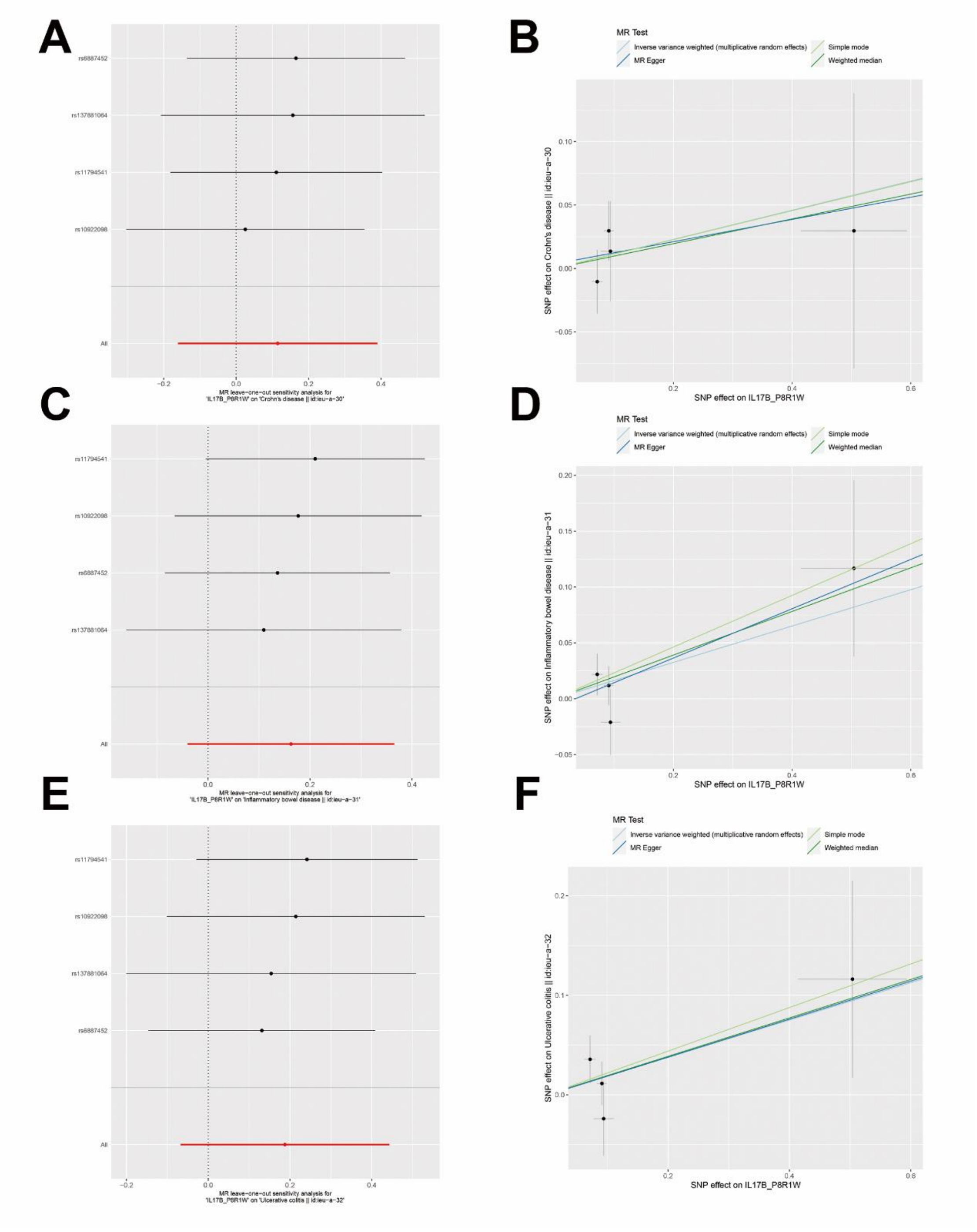

FigureS4. Scatter plot (A-C) of the causal effect of IL-17B on IBD (UC, CD) in OmicSCIENCE dataset.

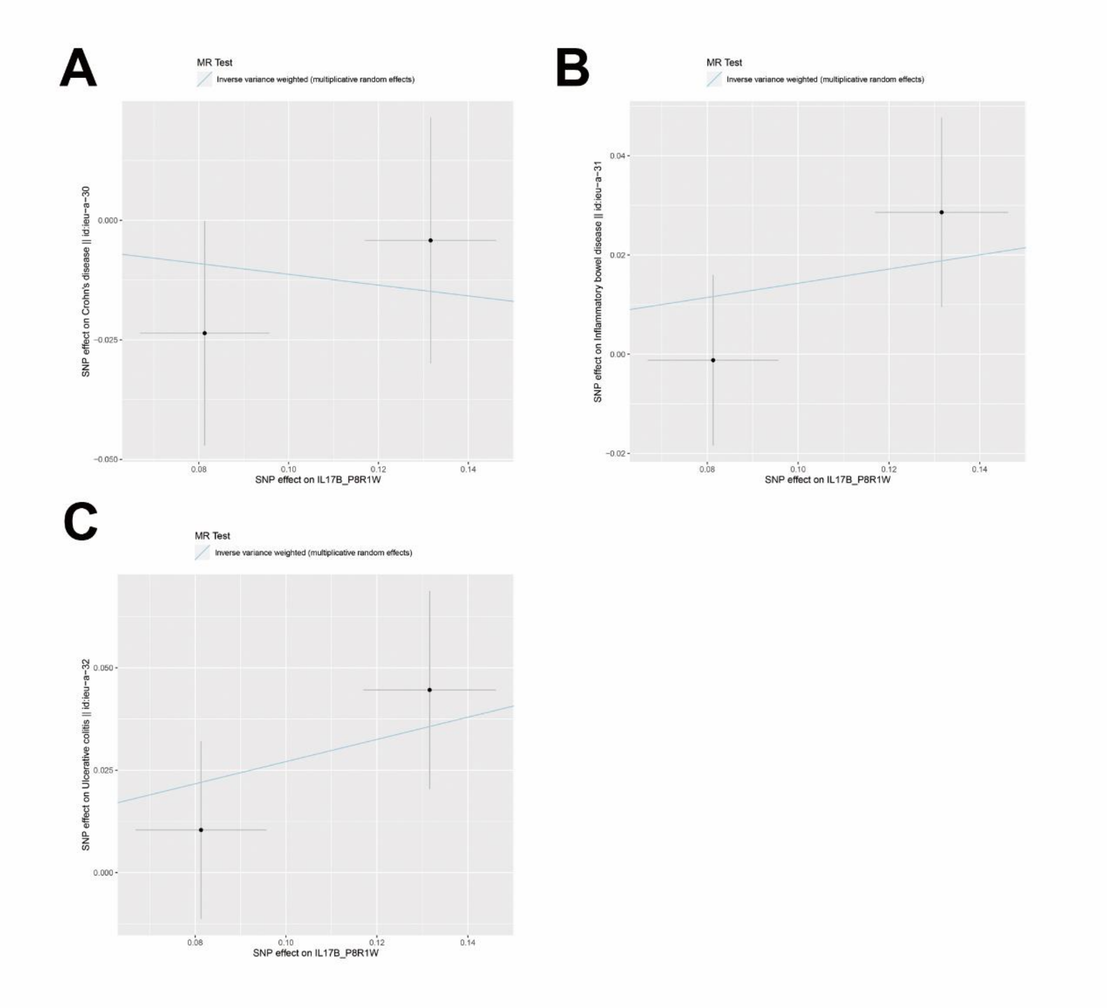

FigureS5. Scatter plot (A-C, D-F) of the causal effect of IL-17C on IBD (UC, CD) in deCODE dataset and OmicSCIENCE dataset, respectively.

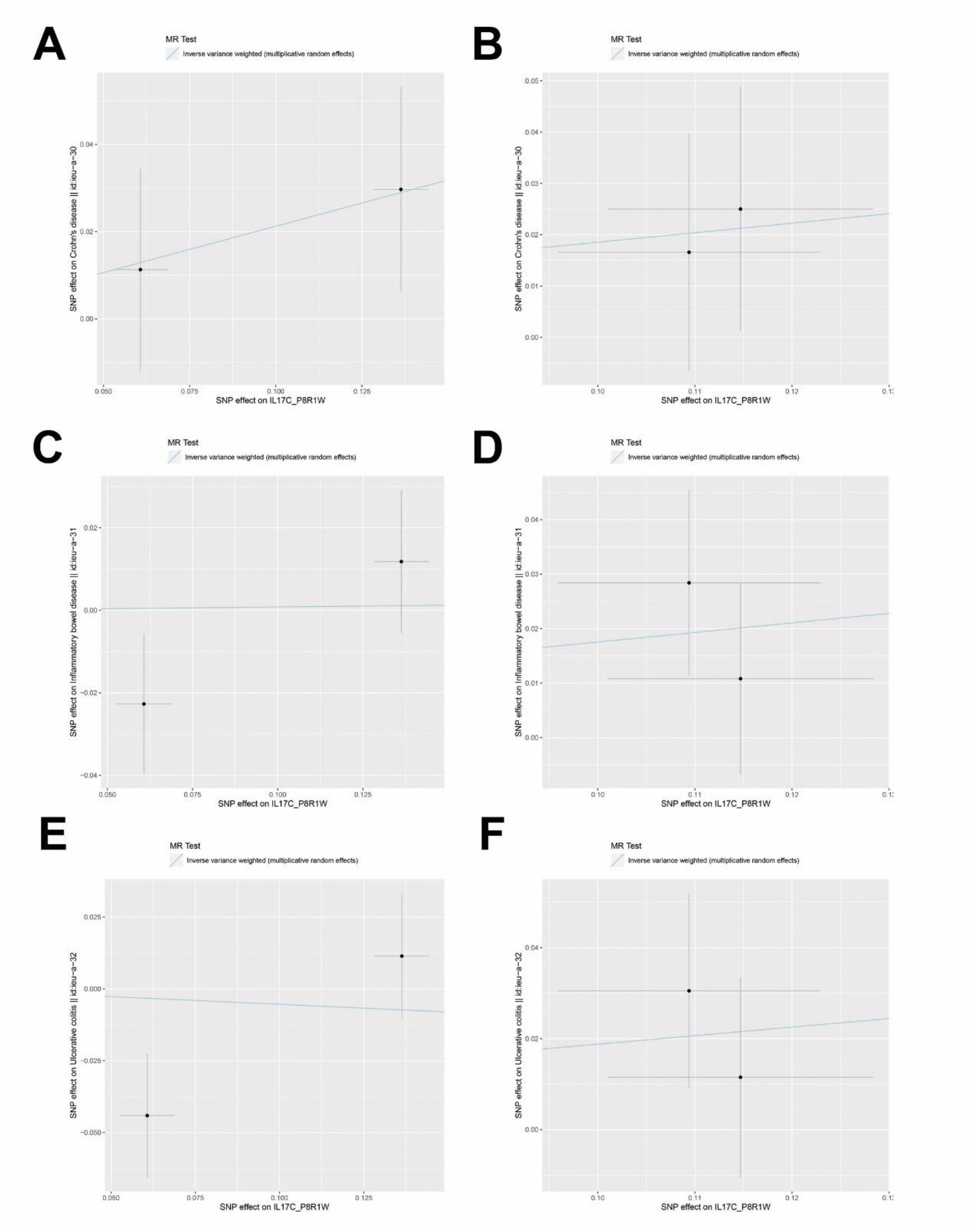

FigureS6. Scatter plot (A-C) of the causal effect of IL-17D on IBD (UC, CD) in OmicSCIENCE dataset.

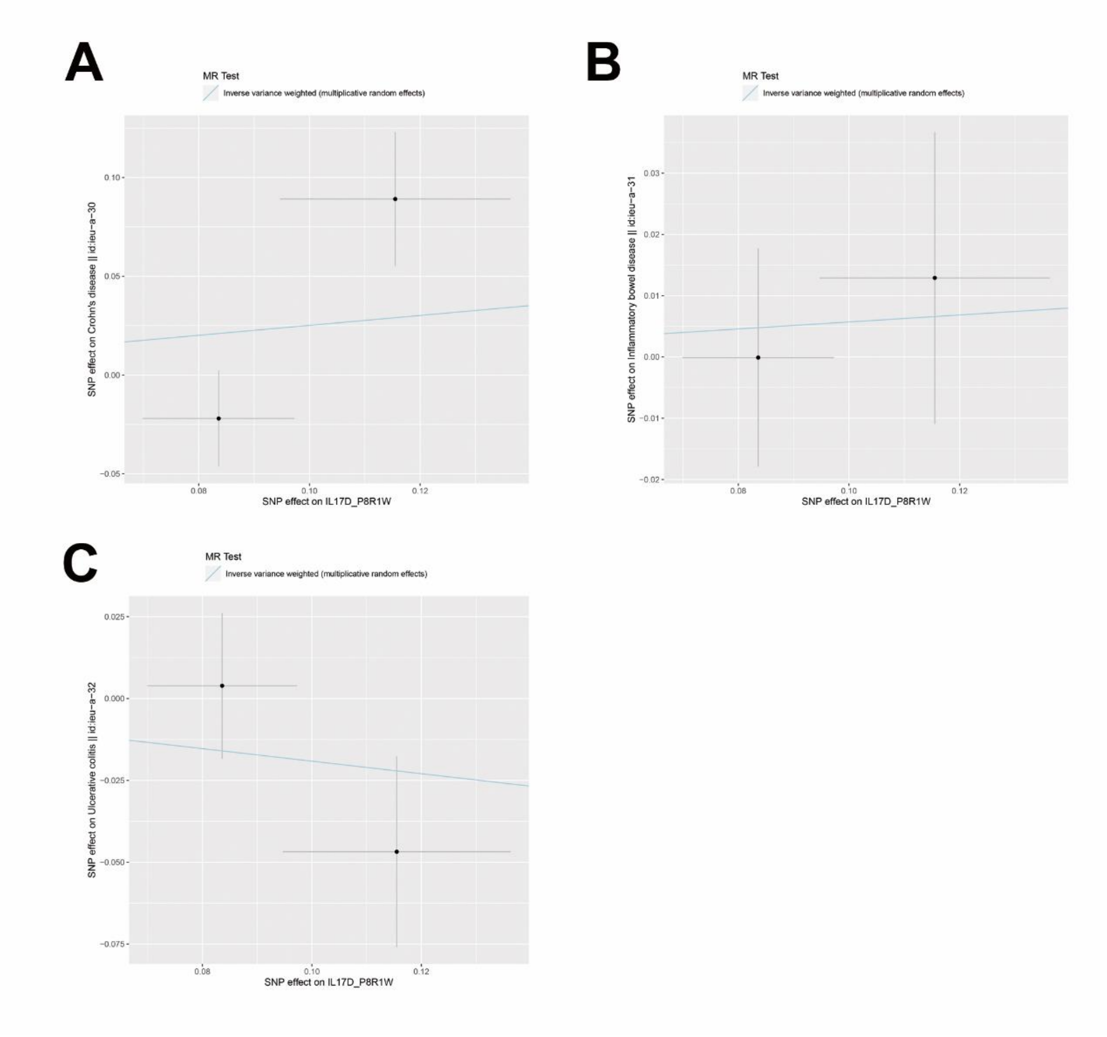

FigureS7. Scatter plot (A-C) of the causal effect of IL-17E on IBD (UC, CD) in deCODE dataset.

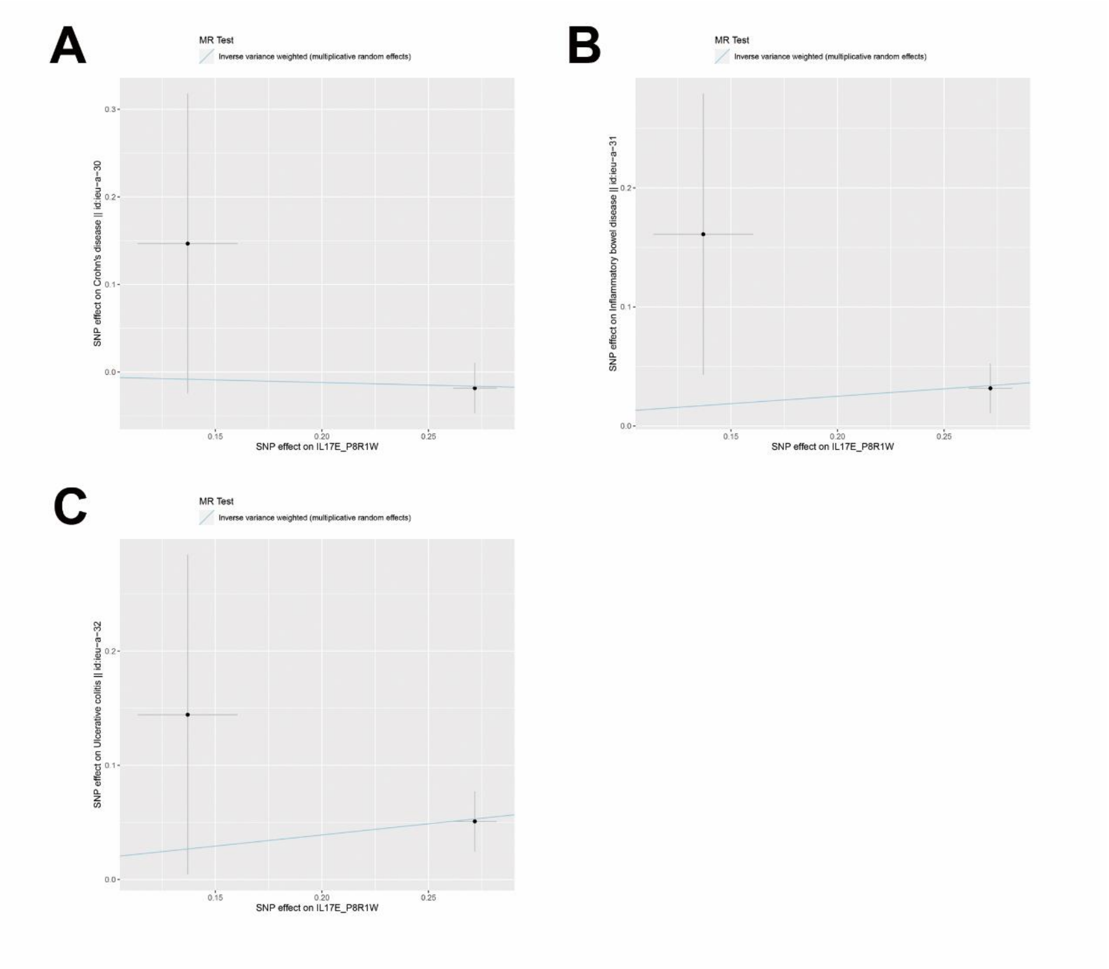

FigureS8. Leave-one-out analysis (A, C, E), and scatter plot (B, D, F) of the causal effect of IL-17E on IBD (UC, CD) in OmicSCIENCE dataset.

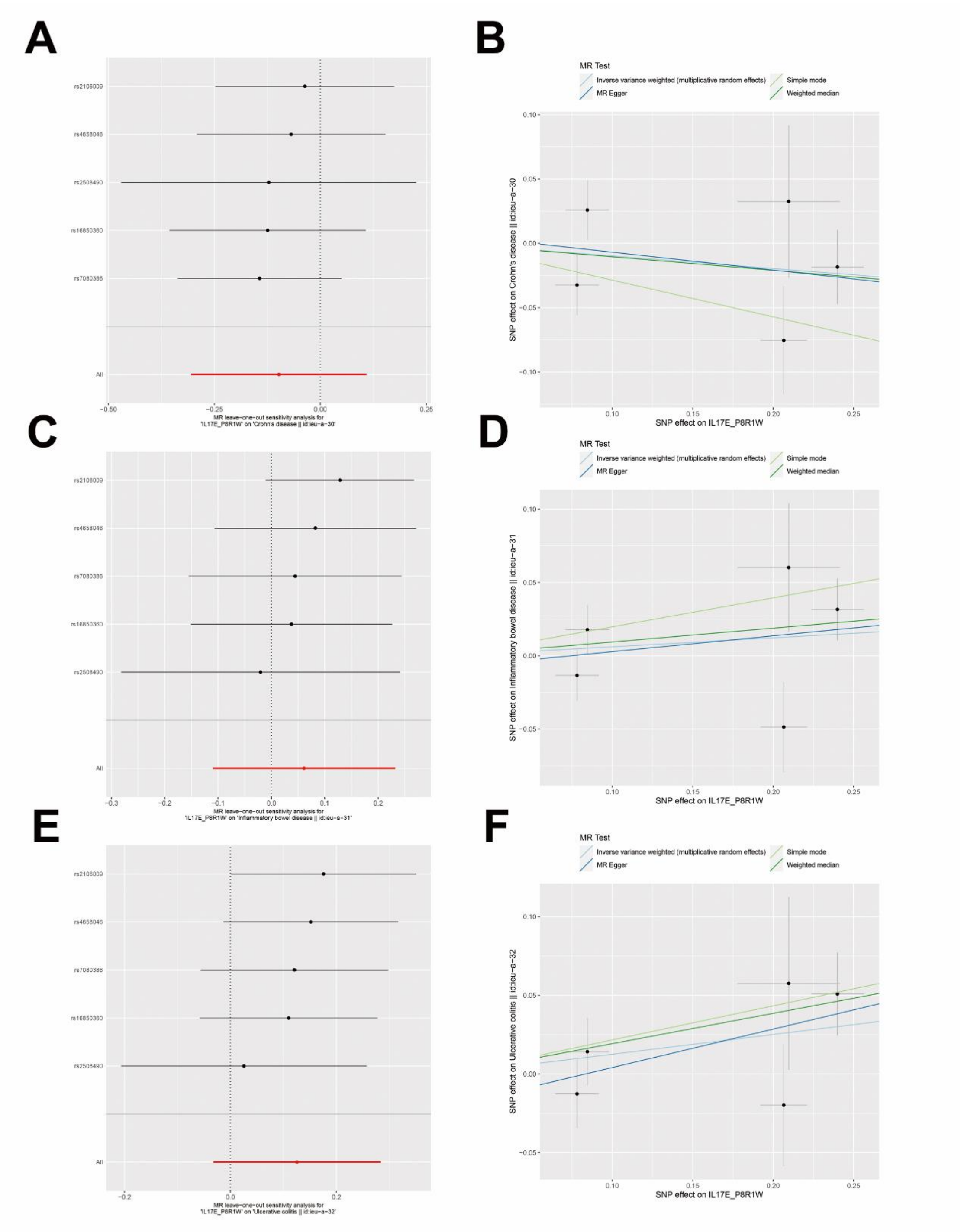

FigureS9. Leave-one-out analysis (A, C, E), and scatter plot (B, D, F) of the causal effect of IL-17F on IBD (UC, CD) in deCODE dataset.

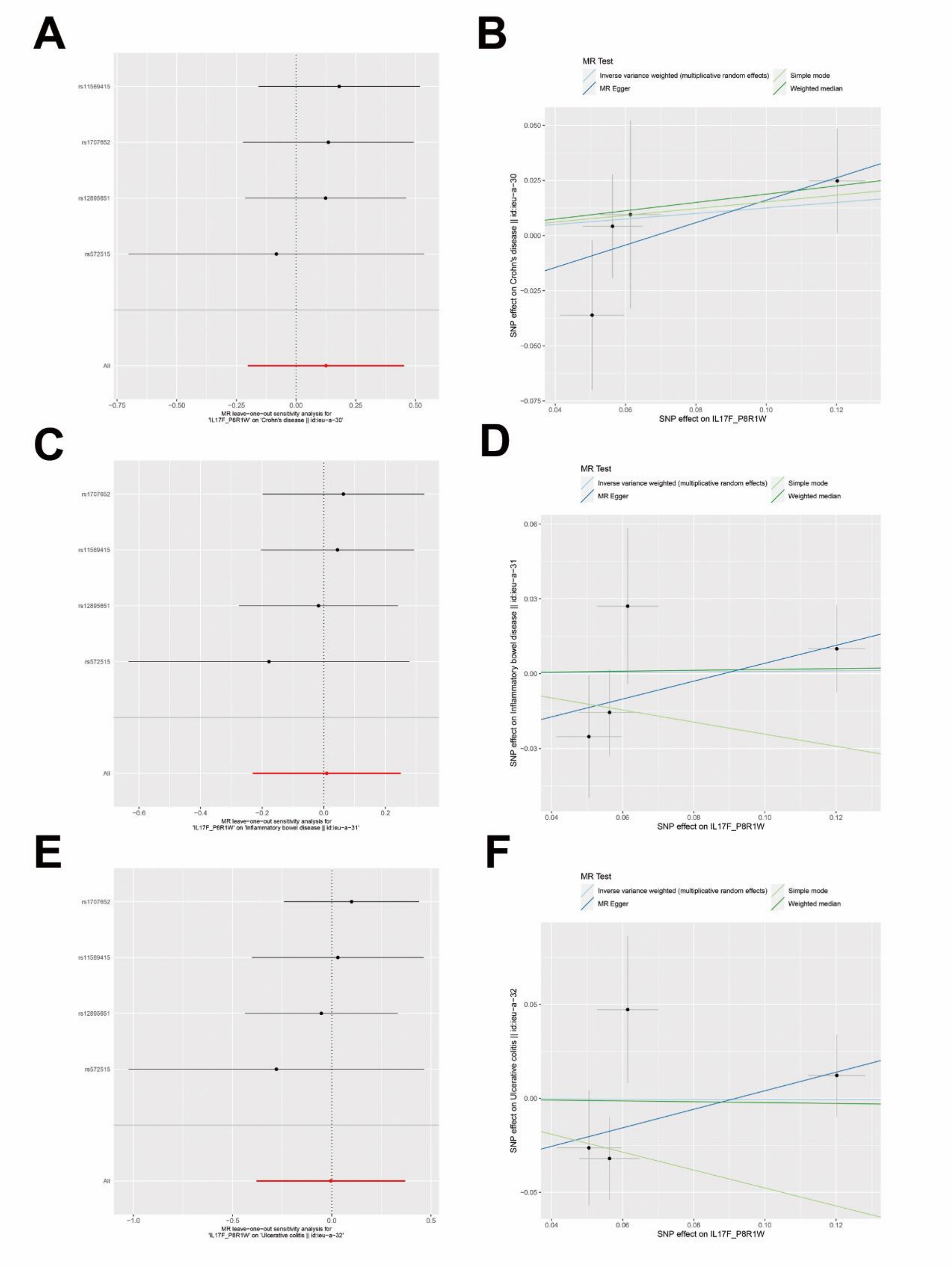

FigureS10. Leave-one-out analysis (A, C, E), and scatter plot (B, D, F) of the causal effect of IL-17F on IBD (UC, CD) in OmicSCIENCE dataset.

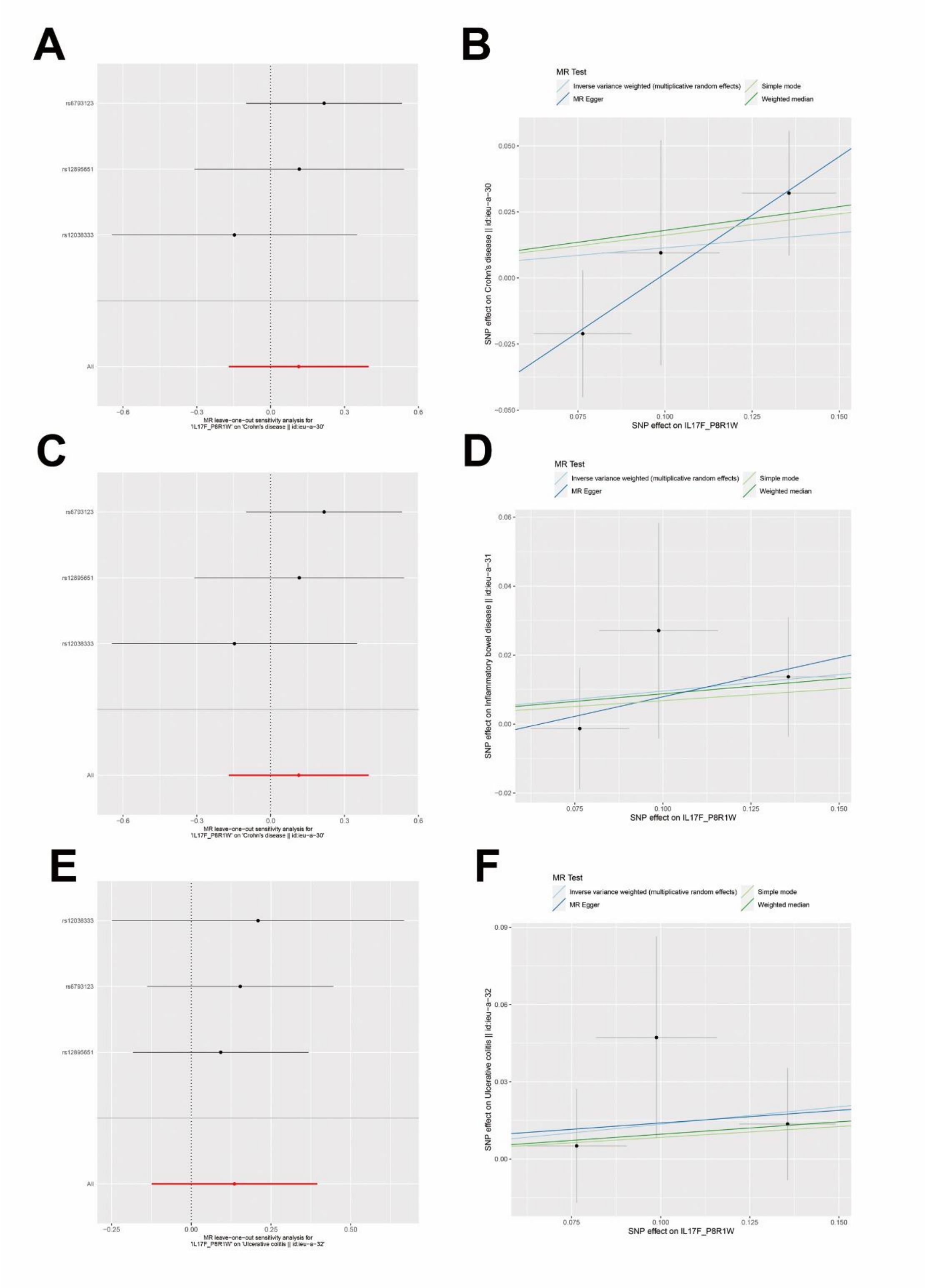

FigureS11. Leave-one-out analysis (A, C, E), and scatter plot (B, D, F) of the causal effect of IL-17RA on IBD (UC, CD) in deCODE dataset.

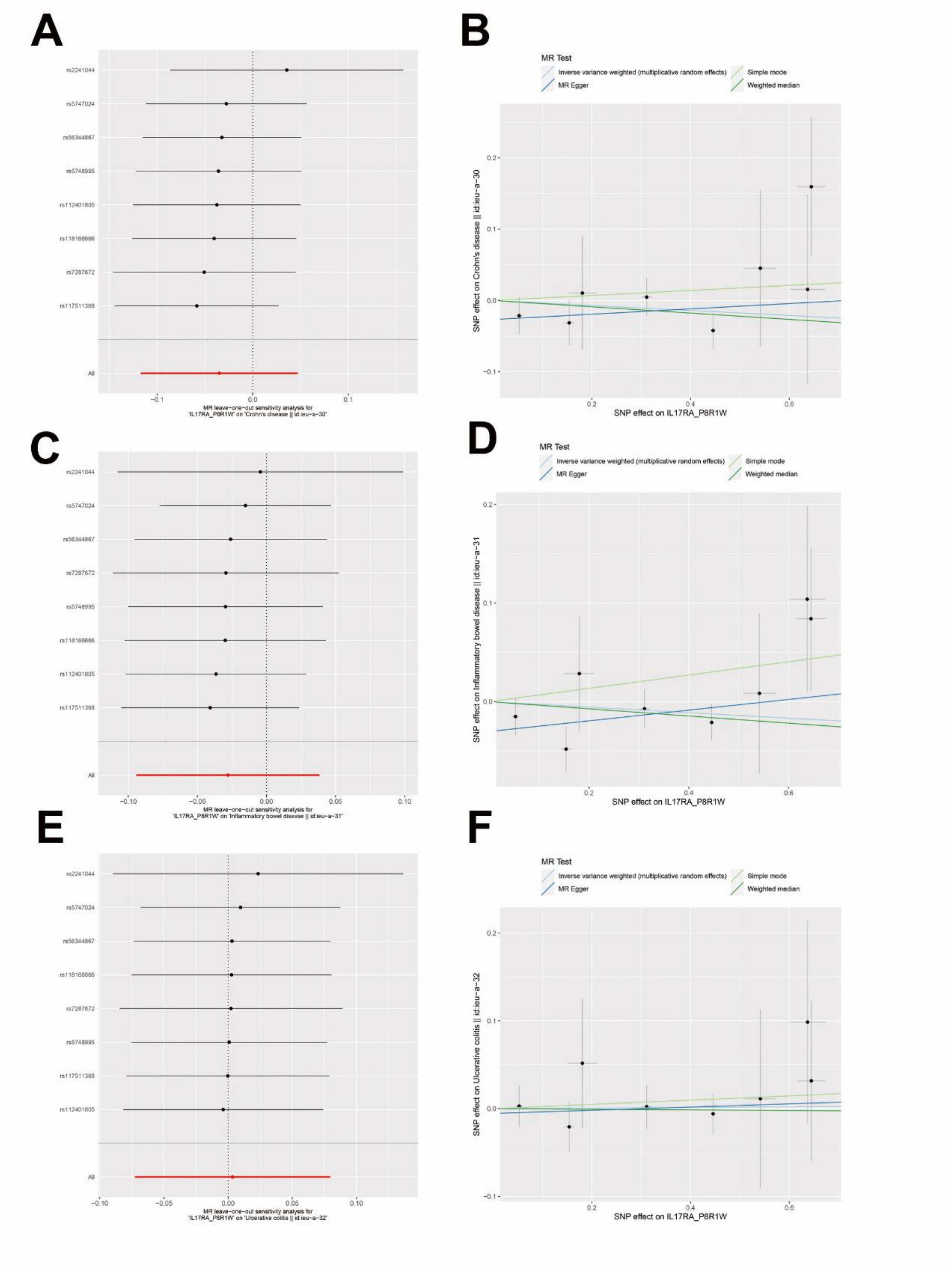

FigureS12. Leave-one-out analysis (A, C, E), and scatter plot (B, D, F) of the causal effect of IL-17RA on IBD (UC, CD) in OmicSCIENCE dataset.

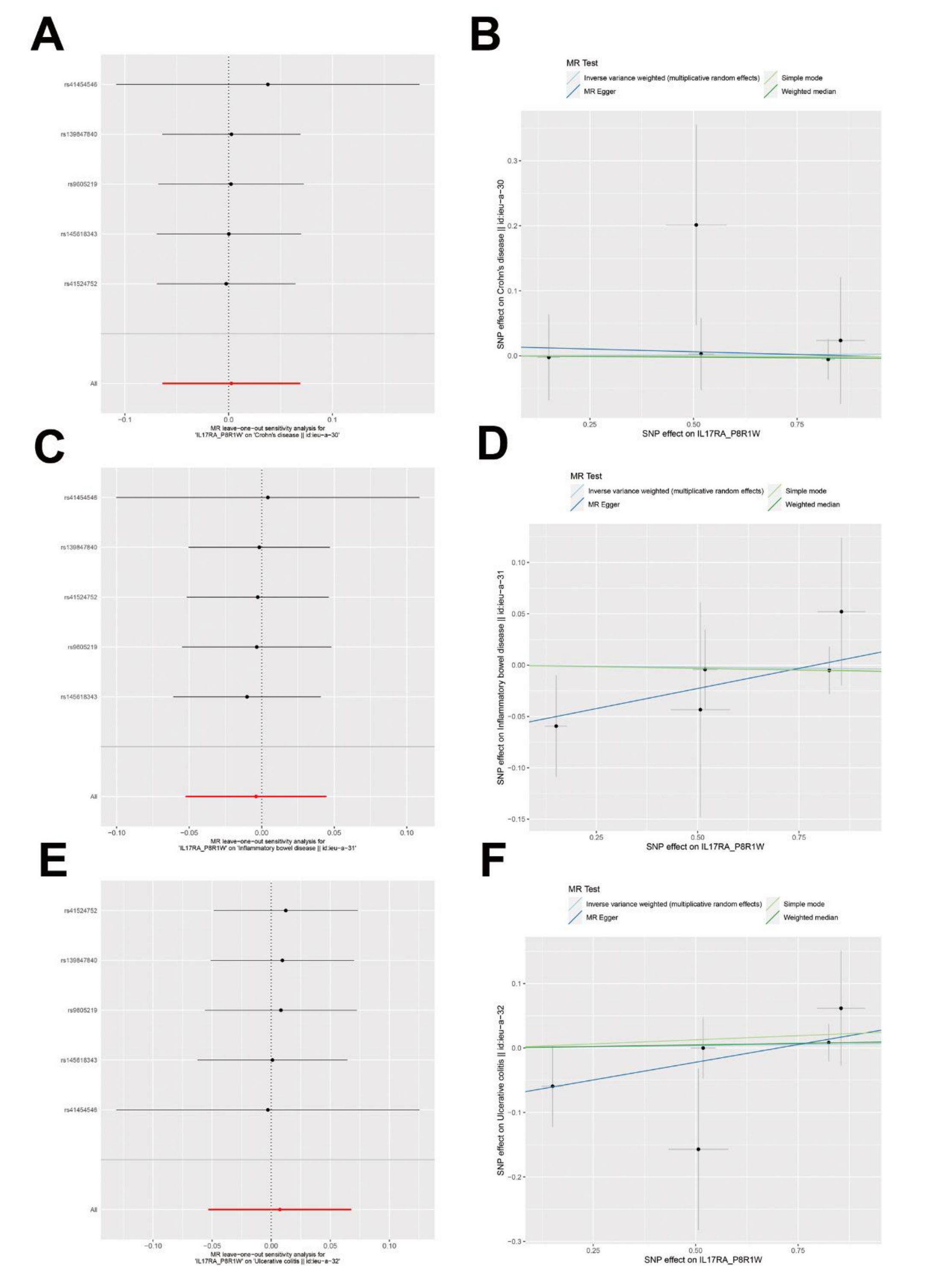

FigureS13. Leave-one-out analysis (A, C, E), and scatter plot (B, D, F) of the causal effect of IL-17RB on IBD (UC, CD) in deCODE dataset.

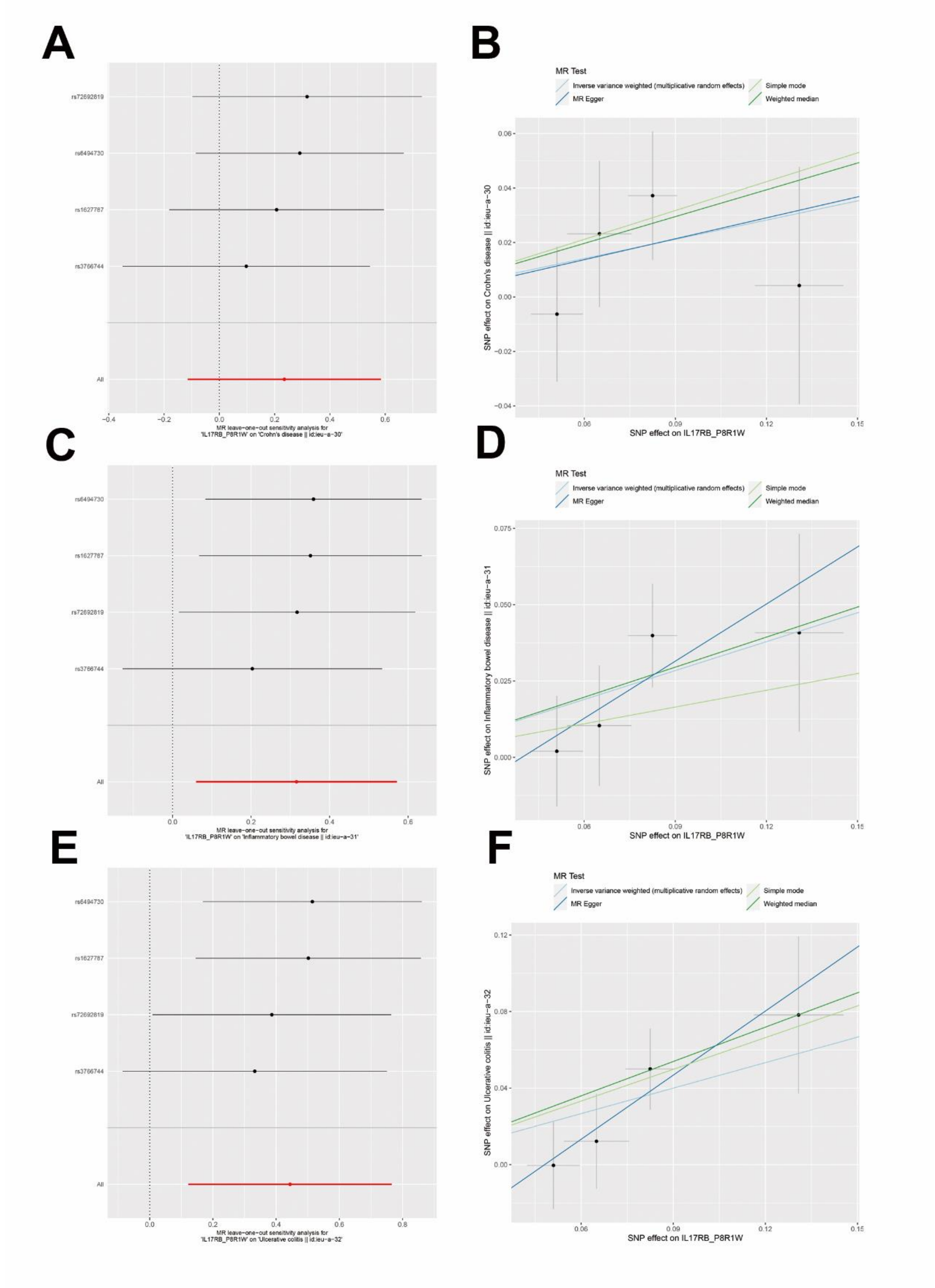

FigureS14. Leave-one-out analysis (A, C, E), and scatter plot (B, D, F) of the causal effect of IL-17RB on IBD (UC, CD) in OmicSCIENCE dataset.

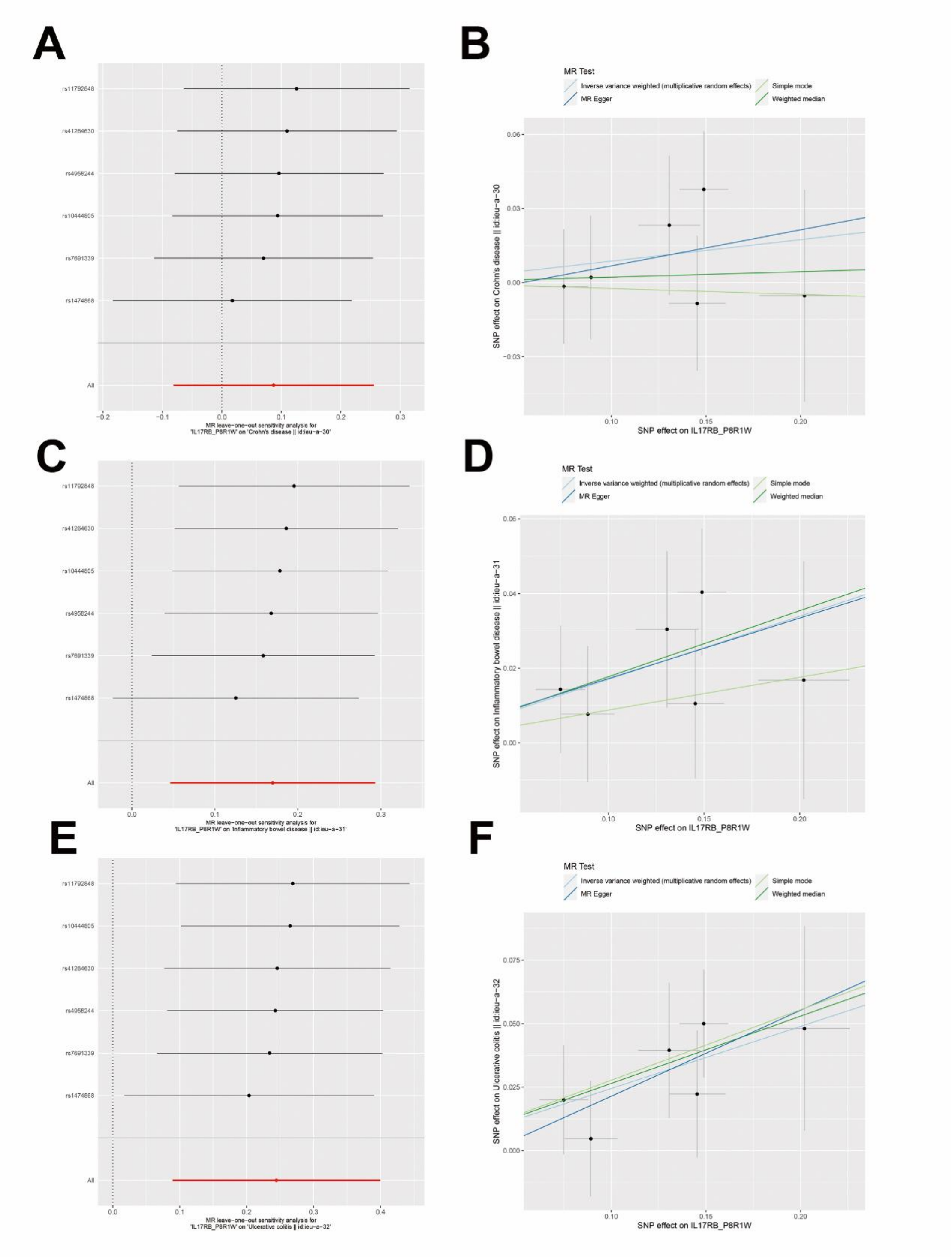

FigureS15. Leave-one-out analysis (A, C, E), and scatter plot (B, D, F) of the causal effect of IL-17RC on IBD (UC, CD) in deCODE dataset.

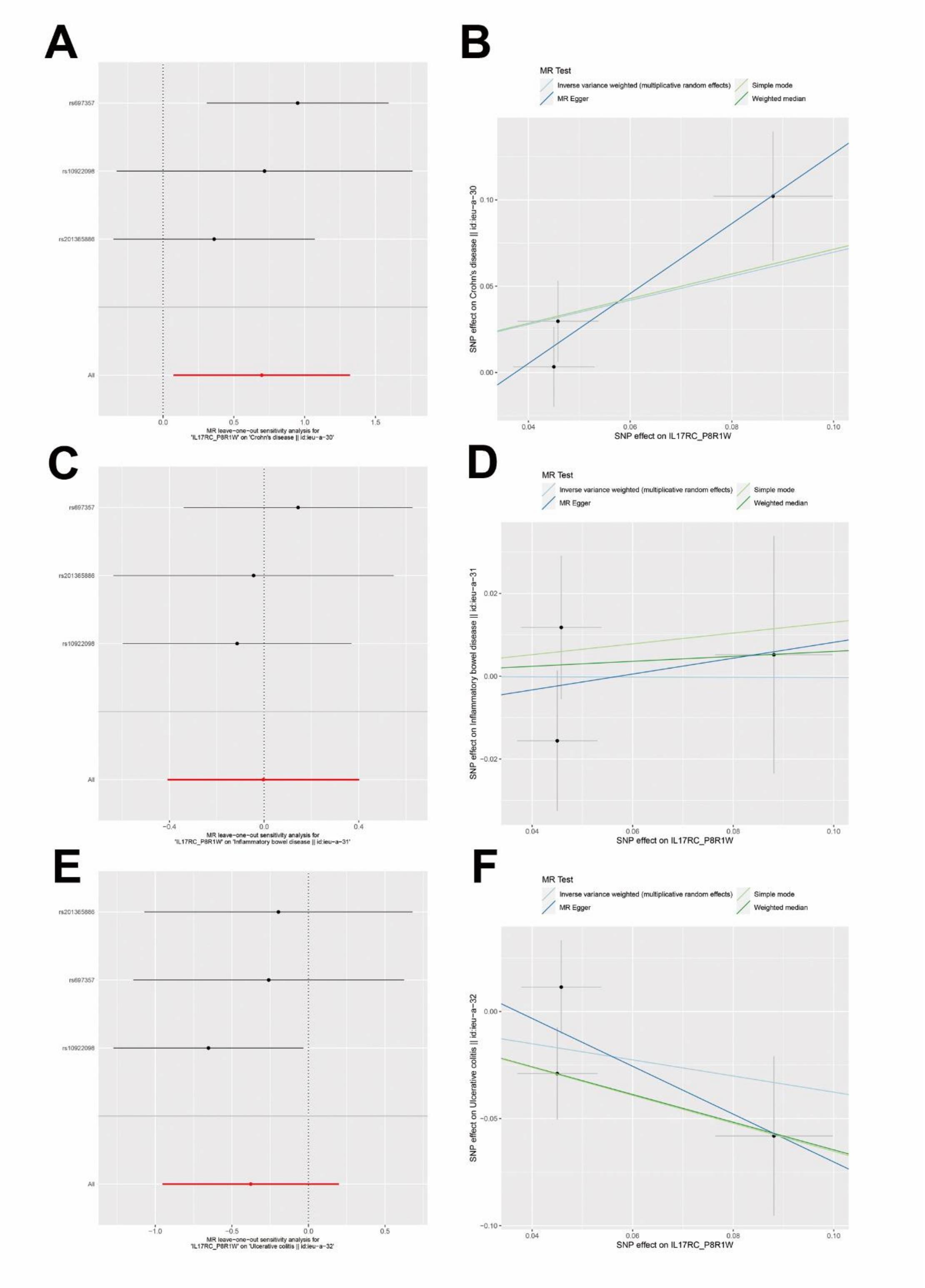

FigureS16. Leave-one-out analysis (A, C, E), and scatter plot (B, D, F) of the causal effect of IL-17RD on IBD (UC, CD) in deCODE dataset.

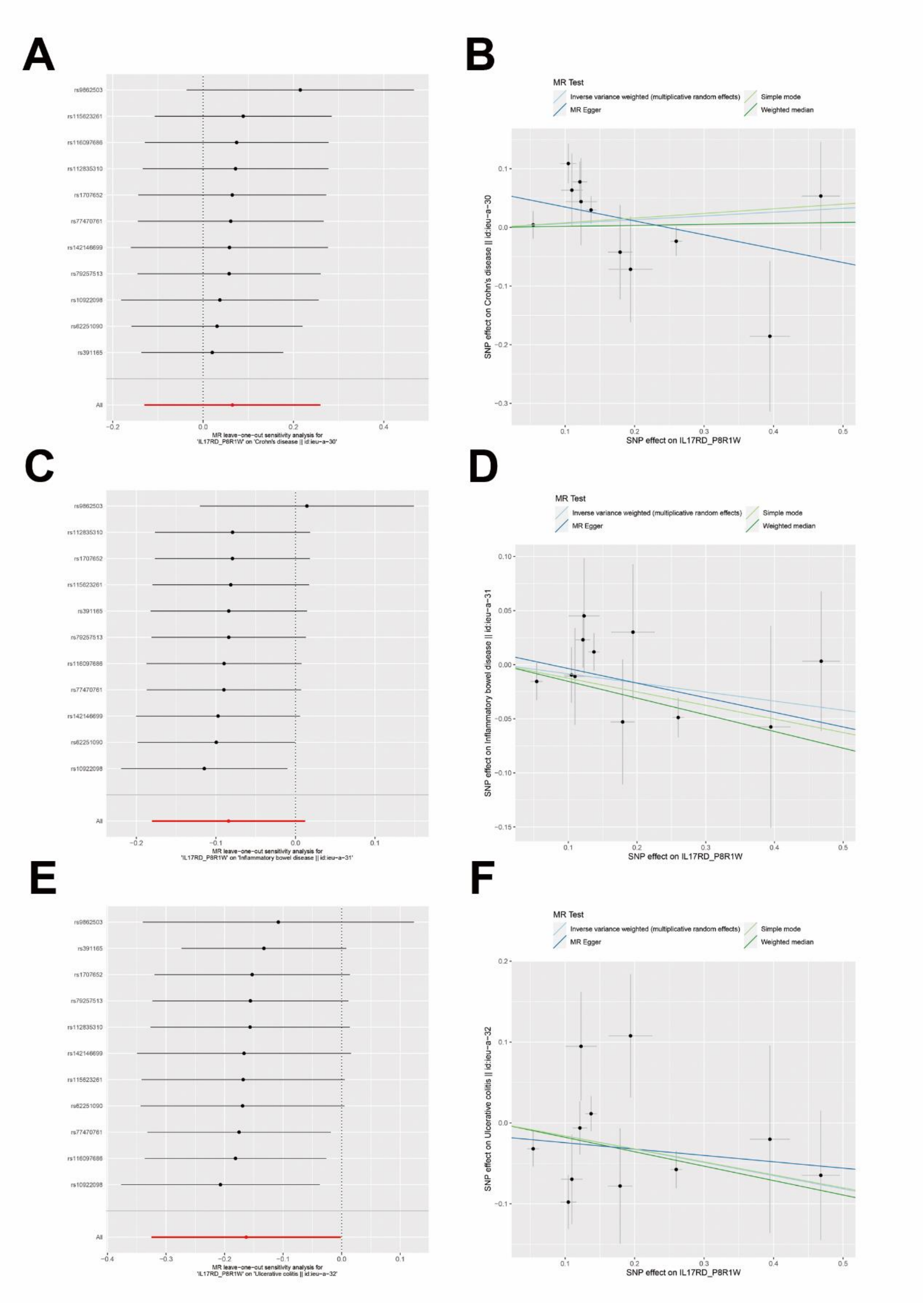

FigureS17. Leave-one-out analysis (A, C, E), and scatter plot (B, D, F) of the causal effect of IL-17RD on IBD (UC, CD) in OmicSCIENCE dataset.

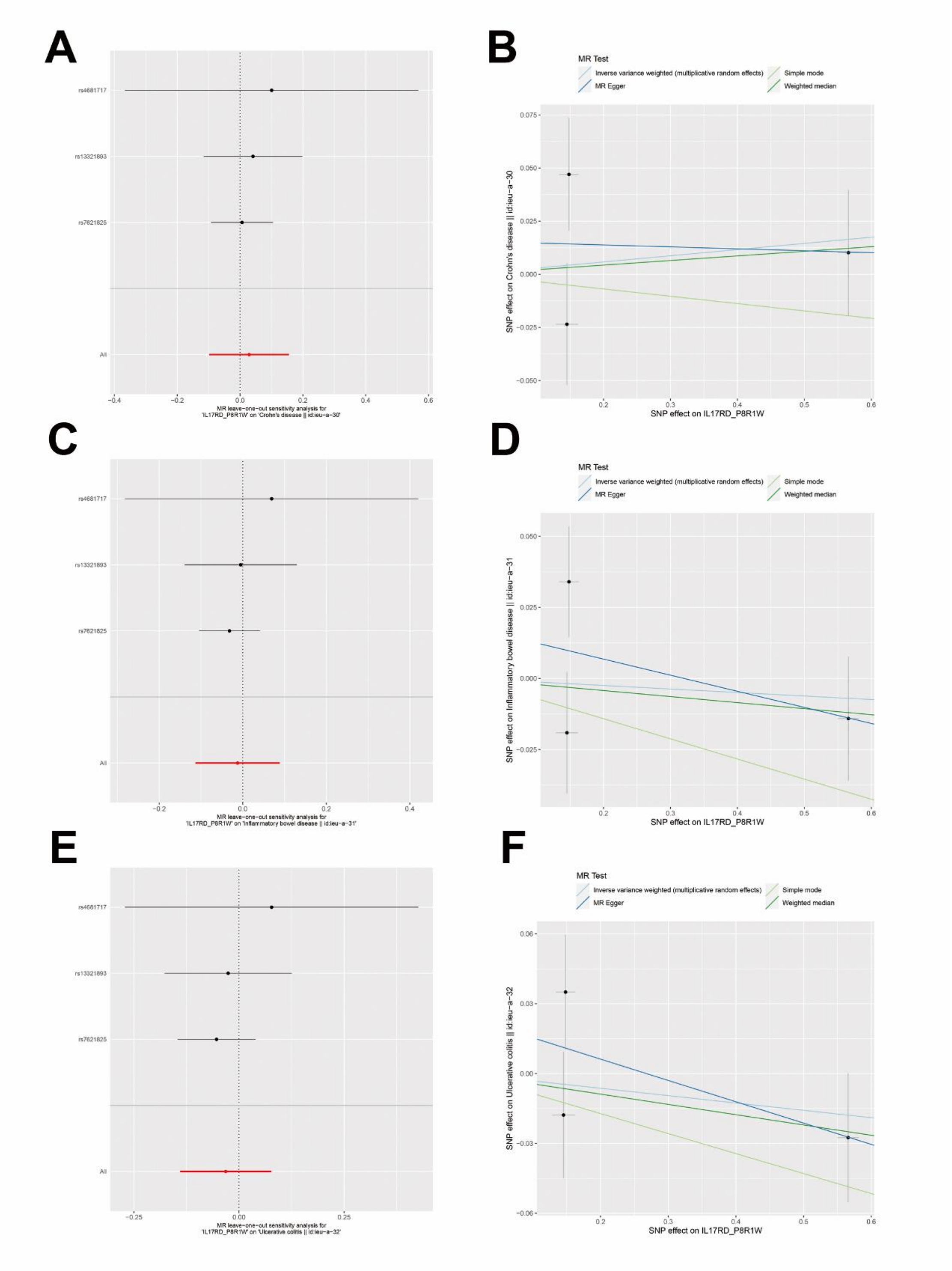

Supplement: Supplementary file 1 [file DataSheet_1.pdf]
